# Using adipose‐derived mesenchymal stem cells to fight the metabolic complications of obesity: Where do we stand?

**DOI:** 10.1111/obr.13413

**Published:** 2022-01-05

**Authors:** Agnieszka Mikłosz, Barbara Emilia Nikitiuk, Adrian Chabowski

**Affiliations:** ^1^ Department of Physiology Medical University of Bialystok Bialystok Poland

**Keywords:** adipose tissue, ADMSCs, metabolic syndrome, obesity

## Abstract

Obesity is a critical risk factor for the development of metabolic diseases, and its prevalence is increasing worldwide. Stem cell‐based therapies have become a promising tool for therapeutic intervention. Among them are adipose‐derived mesenchymal stem cells (ADMSCs), secreting numerous bioactive molecules, like growth factors, cytokines, and chemokines. Their unique features, including immunosuppressive and immunomodulatory properties, make them an ideal candidates for clinical applications. Numerous experimental studies have shown that ADMSCs can improve pancreatic islet cell viability and function, ameliorate hyperglycemia, improve insulin sensitivity, restore liver function, counteract dyslipidemia, lower pro‐inflammatory cytokines, and reduce oxidative stress in the animal models. These results prompted scientists to use ADMSCs clinically. However, up to date, there have been few clinical studies or ongoing trails using ADMSCs to treat metabolic disorders such as type 2 diabetes mellitus (T2DM) or liver cirrhosis. Most human studies have implemented autologous ADMSCs with minimal risk of cellular rejection. Because the functionality of ADMSCs is significantly reduced in subjects with obesity and/or metabolic syndrome, their efficacy is questioned. ADMSCs transplantation may offer a potential therapeutic approach for the treatment of metabolic complications of obesity, but randomized controlled trials are required to establish their safety and efficacy in humans prior to routine clinical use.

**Abbreviations**
ADMSCs: adipose‐derived mesenchymal stem cells
ADMSC‐CM: conditioned medium from ADMSCs
ALT: alanine aminotransferase
AST: aspartate aminotransferase
BAT: brown adipose tissue
BM‐HSC: bone marrow‐derived hematopoietic stem cells
BMI: body mass index
BM‐MSCs: bone marrow mesenchymal stem cells
CAM: cell adhesion molecules
CAT: catalase
CCL18: chemokine ligand‐18
CK‐18: cytokeratin 18
CVD: cardiovascular disease
CXCL9: chemokine (C‐X‐C motif) ligand 9
CXCL10: chemokine (C‐X‐C motif) ligand 9
DIO: diet‐induced obesity
EGF: epidermal growth factor
ERK: extracellular signal regulated kinases
FFAs: free fatty acids
FGF: fibroblast growth factor
G‐CSF: granulocyte colony‐stimulating factor
GLP‐1: glucagon‐like peptide 1
GLUT2: glucose transporter 2
GM‐CSF: granulocyte macrophage colony‐stimulating factor
G6Pase: glucose‐6‐phosphatase
GPx: glutathione peroxidases
GSH: glutathione
GTT: glucose tolerance test
Hb1Ac: glycosylated hemoglobin
HDL‐C: high‐density lipoprotein cholesterol
HF/HSD: high‐fat and high‐sucrose diet
HFD: high‐fat diet
HGF: hepatocyte growth factor
HLCs: hepatocyte‐like cells
HO‐1: heme oxygenase‐1
HOXC10: homeobox C10
ICAs: islet‐like cell aggregates
IDO: indoleamine 2,3‐dioxygenase
IS‐AD‐MSC: adipose‐derived insulin secreting mesenchymal stromal cells
IFN‐γ: interferon γ
IGF‐1: insulin‐like growth factor 1
IKKβ: inhibitory‐κB kinase
IL‐1β: interleukin 1β
IL‐6: interleukin 6
IL‐8: interleukin 8
IL‐1RA: IL‐1 receptor antagonist
IPCs: insulin producing cells
IR: insulin resistance
IRS‐1: insulin receptor substrate 1
JNK: c ‐Jun N‐terminal kinase
LDL‐C: low‐density lipoprotein cholesterol
LDH: lactate dehydrogenase
LIF: leukemia inhibitory factor
LPS: lipopolysaccharide
MAPK: mitogen‐activated protein kinase
MCP‐1, aka CCL2: monocyte chemoattractant protein 1
MIP‐1α, aka CCL3: macrophage inflammatory protein
MIP‐1β, aka CCL4: macrophage inflammatory protein
MHO: metabolically healthy obese
MMP‐2: matrix metalloproteinase‐2
MPO: myeloperoxidase
MS: metabolic syndrome
MSCs: mesenchymal stem cells
mTORC1: mTOR Complex 1
mTORC2: mTOR Complex 2
NAFLD: nonalcoholic fatty liver disease
NASH: nonalcoholic steatohepatitis
NGF: nerve growth factor
NKs: natural killers
Nkx2.2: NK2 homeobox 2
Nkx6.1: NK6 homeobox 1
NO: nitric oxide
NQO1: NAD(P)H quinone oxidoreductase 1
PAI ‐1: plasminogen activator inhibitor‐1
Pax‐6: paired box protein 6
PCOS: polycystic ovary syndrome
PDGF: platelet‐derived growth factor
Pdx‐1: pancreatic and duodenal homeobox gene 1
PEPCK: phosphoenolpyruvate carboxykinase
PGE2: prostaglandin 2
PI3K: phosphatidylinositol 3 kinase
PNPLA3: phospholipase domain‐containing protein 3
PODXL: podocalyxin‐like protein
PPAR‐γ: peroxisome proliferator‐activated receptor gamma
ROS: radical oxygen species
SAT: subcutaneous adipose tissue
S6K1: ribosome protein subunit 6 kinase 1
SISK: stress‐induced serine kinases
SMC: smooth muscle cells
SOD: superoxide dismutase
STAT3: signal transducer and activator transcription 3
STZ: streptozotocin
SVF: stromal vascular fraction
TBIL: total bilirubin level
T1DM: type 1 diabetes mellitus
T2DM: type 2 diabetes mellitus
TBX15: T‐box 15
TC: total cholesterol
TGs: triglycerides
TIMP‐1: tissue inhibitor of metalloproteinases 1
TGF‐β: transforming growth factor β
TNF‐α: tumor necrosis factor α
TSG6: TNF‐inducible gene‐6 protein
UC‐MSCs: umbilical cord‐derived mesenchymal stem cells
UCP‐1: uncoupling protein 1
VAT: visceral adipose tissue
VEGF: vascular endothelial growth factor
WAT: white adipose tissue

## 1 INTRODUCTION

Obesity is a serious global public health problem, responsible for about 4.7 million premature deaths each year.^1^, ^2^ The incidence of obesity is increasing dramatically, as reported by the World Health Organization (WHO); it affected over 650 million adults worldwide in 2016.^3^ Obesity is defined as the excessive accumulation or abnormal distribution of fat tissue.^4^ The current most widely used criteria for classifying obesity is the body mass index (BMI), which ranges from class 1 of obesity (BMI ≥ 30.0 kg/m^2^) to severe or morbid obesity (BMI ≥ 40 kg/m^2^). Obesity can progressively cause and/or exacerbate a wide spectrum of metabolic comorbidities, including type 2 diabetes mellitus (T2DM), hypertension, dyslipidemia, cardiovascular disease (CVD), nonalcoholic fatty liver disease (NAFLD), and fertility problems.^5^, ^6^ The severity and duration of obesity are associated with the metabolic syndrome (MS), which occurs in 4.9% of nonobese patients to 35.3% in patients with obesity.^7^ According to the National Institutes of Health, a subject has MS if it satisfies three or more of the following traits: large waist circumference (≥89 cm for women and ≥102 cm for men), hypertriglyceridemia (≥1.7 mmol/L), reduced high‐density lipoprotein cholesterol (HDL‐C) (<1.04 mmol/L in men or <1.3 mmol/L in women), hypertension (≥130/≥85 mm Hg), and elevated fasting blood glucose (≥5.6 mmol/L).^8^ In light of the alarming data, it is of primary importance to elucidate the mechanism through which obesity leads to the adipose tissue dysfunction followed by metabolic derangements. Nevertheless, many cohort studies have reported that some individuals with obesity remain insulin sensitive and are metabolically “healthy” despite similar total fat mass.^9^, ^10^, ^11^, ^12^ Metabolically healthy obese (MHO) subjects exhibit increased subcutaneous adiposity and are characterized by a lower degree of systemic inflammation, but still they are at a higher risk of cardiovascular complications.^13^, ^14^, ^15^, ^16^ Longitudinal studies provide convincing evidence that MHO is only a transient condition.^17^, ^18^, ^19^ Therefore, it is important to identify individuals with obesity at increased risk of developing obesity‐related metabolic diseases that can benefit most from weight loss. Many factors are responsible for the established obesity‐related disease complications, which lead to a not effective treatment and management of patients with obesity. Recently, numerous strategies have been proposed to minimize health‐related consequences of obesity, including cell‐based therapy. Mesenchymal stem cell (MSC) therapies may represent promising adjunctive therapy for patients with obesity, thus reducing the economic burden of treatment throughout the patient's life.^20^, ^21^, ^22^, ^23^ Both bone marrow‐derived MSC and adipose‐derived MSCs (ADMSCs) have become the most commonly used stem cells for cellular therapy in a variety of human diseases. ADMSCs seem to be superior to other MSCs in many aspects, including ease of isolation, their abundance, and better immunomodulatory properties. Patients may be treated with autologous or allogenic ADMSCs with low risk of cellular rejection. These advantages together with the minimal immunogenicity and high immunoregulatory capacity make them attractive for clinical use. In this review, we outline the current understanding of ADMSCs—based therapies in obesity and its associated diseases from the animal model to the preclinical and clinical trials.

## 2 ADIPOSE TISSUE

Adipose tissue was historically considered to be merely an energy store, but this concept was changed after the discovery of leptin in 1990 by Friedman's group.^24^ Since this discovery, other cytokines, hormones, and peptides, collectively referred to as “adipokines,” have been identified.^25^ Adipose tissue develops extensively in homeothermic organisms, and the proportions to body weight vary considerably between species. Averagely, it constitutes about 15%–20% of body mass of men and 20%–25% of women. This connective tissue influences the whole body as it is responsible for energy storage and distribution, fat accumulation, thermoregulation, hormone synthesis, glucose, and insulin homeostasis.^26^, ^27^ Fat tissue can be classified into brown adipose tissue (BAT) and white adipose tissue (WAT), which differ in function, distribution and morphology.^28^ In adult humans, BAT had long been considered to be absent; however, recent investigations have shown that BAT is found to be distributed throughout the cervical, supraclavicular, mediastinal, suprarenal, and paravertebral regions.^29^ The mitochondrial abundance and high vascularization in comparison with the WAT give it a brown color appearance. High expression of uncoupling protein 1 (UCP1) in their inner mitochondrial membrane is responsible for energy dissipation in the process called nonshivering thermogenesis.^30^ In turn, white adipocytes not only control energy balance by storing and mobilizing triacylglycerols but also secrete a variable amount of hormones and paracrine factors. Although white adipocytes are distributed throughout the body, their principal depots are the subcutaneous adipose tissue (SAT) and visceral adipose tissue (VAT).^31^ SAT is found beneath the skin, some deposits are gluteal, femoral, and abdominal, while visceral fat surrounds internal organs and is concentrated in the abdominal cavity, further subdivided into mesenteric, omental, perirenal, and pertoneal depots. Importantly, WAT depots are functionally distinct, SAT stores excess lipid, and thus preventing ectopic lipid deposition, while VAT protects the visceral organs. Typically, VAT can be identified by a higher number of smaller adipocyte size, whereas SAT by larger adipocytes. In healthy middle‐aged adults, only 5%–15% of total body fat is considered VAT; the rest is SAT, the largest body fat deposit. When the storage capacity of adipocytes exceeds (like in obesity), further caloric overload leads to the expansion of adipose tissue in a given fat compartment through increase in adipocyte size (hypertrophy) and/or proliferation of precursor cells (hyperplasia).^29^, ^32^ Simultaneously, the precursor cells of the stromal vascular fraction (SVF) in adipose tissue undergo numerous functional changes, begin to be recruited and committed towards adipocyte lineage. This series of events is called “adipose tissue remodeling.”^33^ However, in obesity, aberrant adipose tissue remodeling may induce dysregulation of fat tissue in secreted cytokines, hormones, and metabolites.^34^ This causes ectopic lipid deposition in the liver, skeletal muscle, heart, pancreas, as well as in the visceral depots and leads to impaired glucose and lipid metabolism, systemic insulin resistance (IR), an increased risk of T2DM and CVD development.^35^, ^36^ It is worth nothing that the distribution of adipose tissue appears to be more important than the total amount of the body fat. Indeed, VAT is more metabolically active, has higher free fatty acids (FFAs) and glucose uptake, is less insulin sensitive, and therefore is thought to be more deleterious in the development of obesity‐related metabolic complications.^37^ In accordance with this statement, Tran et al. transplanted either visceral (intra‐abdominal) or subcutaneous fat from donor to visceral or subcutaneous regions of recipient mice.^38^ Surprisingly, transplantation of SAT in an intra‐abdominal site improves glucose tolerance and the whole‐body insulin sensitivity, suggesting that adipose tissue depots maintain an intrinsic memory of their site of origin and thus have distinct metabolic properties. Similarly, Satoor et al. have shown that autologous transplantation of visceral fat (intra‐abdominal) to subcutaneous (thigh/chest) sites provides metabolic advantage at physiological as well as at molecular levels.^39^ Three weeks after transplantation, the abundance of adipokine gene transcript (i.e., adiponectin, leptin, visfatin, and resistin) was adjusted to the expression level in the resident (thigh) depot. These observations support the notion that adipose tissue depots have “residence memory” and local factors, such as glucose levels, are involved in the epigenetic regulation of adipokine gene promoters.^39^

## 3 MESENCHYMAL STEM CELLS

MSCs also referred to as “mesenchymal stromal cells” are fibroblast‐like multipotent cells characterized by the capacity of self‐renewal and ability to differentiate into cell types of mesodermal origin, including adipocytes, chondrocytes, and osteoblasts. Stem cell research has advanced considerably since pluripotent cells were first isolated from mouse embryos in 1981^40^; however, the first report with embryonic stem cell lines derived from human blastocysts was published in 1998.^41^ The clinical relevance of MSCs was initially based on harnessing their potential for tissue regeneration and repair, and the discovery of their paracrine properties has greatly expanded the range of therapeutic applications for which they are currently being explored. MSCs are attractive cell therapy agents in the treatment of various diseases, especially in the treatment of conditions involving autoimmune and inflammatory processes. Several characteristics favor their use in a wide range of diseases, such as their autocrine and paracrine activities, immunomodulatory and immunosuppressive properties, with their minimal immunogenicity and ethical restrictions.^42^ The cells can be obtained from human's multiple organs and structures like bone marrow, adipose tissue, liver, pancreas, spleen, thymus, skeletal muscle, dental pulp, dermis, and neonatal tissues (umbilical cord, amniotic fluid, fetus, placenta), but the most frequently used sources of MSCs remain bone marrow and adipose tissue.^43^ To standardize MSCs, in 2006, the International Society for Cell and Gene Therapy (ISCT) proposed the following minimal criteria: (1) They must be plastic adherent when maintained in standard culture conditions; (2) they must express the surface markers CD73, CD90, and CD105 and lack of expression of hematopoietic and endothelial antigens CD14 (or CD11b), CD19 (or CD79α), CD34, CD45, and HLA‐DR surface markers; (3) they must be able to differentiate into adipocytes, chondrocytes, and osteocytes in vitro (trilineage potential).^44^

### 3.1 Adipose‐derived MSCs

**FIGURE 1:**
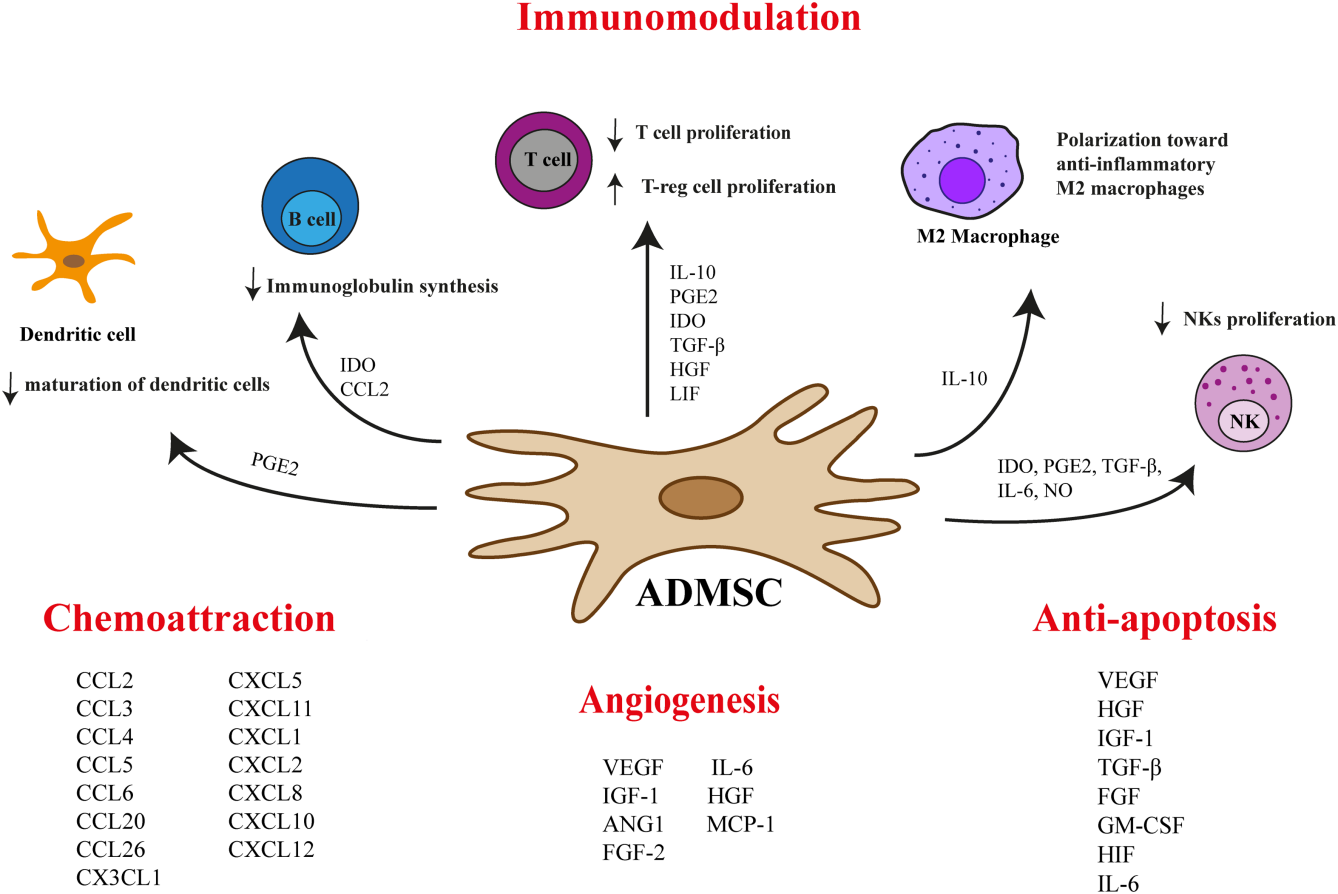
Paracrine functions of ADMSCs. It is now believed that the therapeutic effects of ADMSCs are due to their ability to secrete a wide range of bioactive molecules, including cytokines, chemokines, antioxidant factors, and growth factors. The paracrine mechanism plays a major role in immunomodulation, limitation of apoptosis, and stimulation of local angiogenesis. The immunomodulatory activity of ADMSCs consists of inhibition of dendritic cells (DCs) differentiation, suppression of immunoglobulin synthesis, inhibition of the CD8+ and CD4+ T lymphocytes and natural killer (NK) cells proliferation, and promotion of M2 macrophage polarization and regulatory T cells (Treg) proliferation. Abbreviations: ADMSCs, adipose‐derived mesenchymal stem cells; ANG1, angiopoietin‐1; CCL2, chemokine (C‐C motif) ligand 2; CCL20, chemokine (C‐C motif) ligand 20; CCL26, chemokine (C‐C motif) ligand 26; CCL3, chemokine (C‐C motif) ligand 3; CCL4, chemokine (C‐C motif) ligand 4; CCL5, chemokine (C‐C motif) ligand 5; CCL6, chemokine (C‐C motif) ligand 6; CX3CL1, chemokine (C‐X3‐C motif) ligand 1; CXCL1, chemokine (C‐X‐C motif) ligand 1; CXCL10, chemokine (C‐X‐C motif) ligand 10; CXCL11, chemokine (C‐X‐C motif) ligand 11; CXCL12, chemokine (C‐X‐C motif) ligand 12; CXCL2, chemokine (C‐X‐C motif) ligand 2; CXCL5, chemokine (C‐X‐C motif) ligand 5; CXCL8, chemokine (C‐X‐C motif) ligand 8; FGF, fibroblast growth factor; GM‐CSF, granulocyte macrophage colony‐stimulating factor; HGF, hepatocyte growth factor; HIF, hypoxia inducible factor; IDO, indoleamine 2,3‐dioxygenase; IGF‐1, insulin‐like growth factor 1; IL‐10, interleukin 10; IL‐6, interleukin 6; LIF, leukemia inhibitory factor; MCP‐1, monocyte chemoattractant protein 1; NK, natural killer cells; NO, nitric oxide; PGE2, prostaglandin 2; TGF‐β, tumor growth factor β; VEGF, vascular endothelial growth factor

ADMSCs hold great promise as a therapeutic strategy in treating a wide spectrum of diseases like obesity, T2DM, fatty liver disease (NAFLD, nonalcoholic steatohepatitis [NASH], liver fibrosis, cirrhosis), CVDs, muscular dystrophy, osteoarthritis, Crohn's disease, cancers, multiple sclerosis, acute kidney injury, and chronic skin wounds.^45^, ^46^, ^47^, ^48^, ^49^, ^50^, ^51^, ^52^, ^53^ ADMSC‐based clinical trials have grown over the years largely due to their abundance, ease of isolation, rapid expansion, high proliferation capacity, and no ethical issues.^54^ The secretion of a broad range of paracrine factors, including cytokines, antioxidant factors, and growth factors, into their microenvironment is believed to be a primary mechanism by which ADMSCs achieve their therapeutic effect (Figure 1). ADMSCs show the typical characteristics of MSCs, after in vitro stimulation can differentiate into mesodermal lineages cell types (adipocytes, osteoblasts, chondrocytes, fibroblasts, and myocytes) as well as no mesodermal cell types, such as neurons, hepatocytes, endothelial cells, and cardiomyocytes.^55^ According to the standard criteria, cultured ADMSCs are plastic‐adherent, spindle‐shape cells characterized by the expression of positive markers: CD13, CD29, CD44, CD73, CD90, and CD105 and the lack of CD45 and CD31 on their surface. Moreover, the characterization of ADMSCs includes additional positive markers like CD10, CD26, CD36, CD49d, and CD49c and low or negative markers like CD3, CD11b, CD49f, CD106, and podocalyxin‐like protein (PODXL).^56^ ADMSCs constitute up to 2% of SVF, a heterogeneous mesenchymal population of cells, compared with the low cell yield (0.001%–0.002%) of BM‐MSCs. A large amount of ADMSCs is isolated from SAT by liposuction or fat excision with an efficiency up to 500 times greater than from bone marrow isolation. The liposuction procedure provides 100 ml to 3 L of lipoaspirate that is routinely discarded. By processing this material, ADMSCs are isolated from the SVF, yielding up to six billion cells in one passage.^54^, ^57^ Some features of ADMSCs are similar to BM‐MSCs, but numerous properties are different. For instance, ADMSCs are more susceptible to differentiate into pancreatic beta cells, muscle cells, and cardiomyocytes compared with BM‐MSCS. In addition, some studies reported a greater osteogenic capacity of BM‐MSC than of ADMSC^58^, ^59^, ^60^; however, other studies have shown equal or even superior osteogenic capacity of ADMSC,^61^, ^62^ making them suitable for bone tissue engineering. Furthermore, phenotypic expression patterns allow to distinguish between stem cell derived from adipose tissue or bone marrow; that is, only ADMSCs express CD36 and CD49d, whereas CD106 antigen is expressed solely by BM‐MSCs. Recent evidence indicates that ADMSCs are stronger immunomodulators and are better adapted to oxidative stress, hypoxia‐induced apoptosis or have a greater angiogenetic force when exposed to harsh conditions compared with BM‐MSCs.^63^ Their advantage in immune regulation is due to the secretion of higher levels of pro‐inflammatory and anti‐inflammatory cytokines such as interleukins (IL‐6, IL‐8), interferon γ (IFN‐γ), and transforming growth factor (TGF‐β). ADMSCs also release higher amount of growth factors including granulocyte colony‐stimulating factor (G‐CSF), granulocyte macrophage colony‐stimulating factor (GM‐CSF), nerve growth factor (NGF), or insulin‐like growth factor 1 (IGF‐1) compared with the BM‐MSCc.^64^, ^65^, ^66^, ^67^ Overall, the superior characteristics of ADMSCs compared with other MSCs along with their abundance and easy cell access to cells encourage scientists to complete researchers among ADMSCs.

In recent years, it has been proven that there are differences between ADMSCs isolated form lean and individuals with obesity. Approximately 10% of adipocytes are renewed annually at all BMI levels; however, in subjects with obesity, an excess of adipocyte generation results from an increased abilities of ADMSCs to differentiate to adipocyte lineage.^68^ ADMSCs collected from morbidly obese patients exhibit diminished expression of two fundamental developmental transcription factors, that is, T‐box 15 (TBX15) and the homeobox C10 (HOXC10), and ACTA2, a marker of ADMSCs. Downregulation of these factors indicates that obesity interferes with ADMSCs multipotency, especially in obese patients with MS. On the contrary, the inflammatory genes IL‐1β and IL‐8 and monocyte chemoattractant protein 1 (MCP‐1, aka CCL2) were upregulated in ADMSCs acquired from individuals with obesity.^69^ This significant release of inflammatory cytokines by ADMSC is associated with the development of low‐grade chronic systemic inflammation during obesity progression. Summing up, ADMSCs isolated from patients with obesity and MS have a lower proliferative and differentiation capacity and therefore are less effective in immunomodulation compared with lean, metabolically healthy individuals. This knowledge is of great interest as ADMSCs have been used in various preclinical models and clinical trials, especially in choosing the adequate ADMSCs subpopulation for therapies.

## 4 RESEARCH AMONG ADMSCS USAGE IN THE TREATMENT OF OBESITY‐RELATED MORBIDITIES

Obesity is a complex, multifactorial disease. Usually excessive fat deposition in obesity is closely related to environmental factors such as an increased consumption of saturated fats, carbohydrates, sugars, and decreased physical activity as well as to genetic and epigenetic factors.^70^ Consumption of excess nutrients causes fat to accumulate in deposits of WAT (subcutaneous and visceral), leading to adipocyte hypertrophy and systemic metabolic dysfunction. Appropriate weight loss is the cornerstone of obesity treatment and should be promptly offered to patients with obesity to prevent and/or delay the onset of obesity‐related complications. Throughout the past half century, a variety of interventions have been proposed for management of obesity. ADMSCs therapy is gaining more and more attention as an attractive strategy for obesity and related comorbidities. The results obtained in in vivo animal models confirmed their therapeutic potential in weight loss and changes in the composition of adipose tissue. Jaber et al. investigated the effect of ADMSCs on body weight and composition in mouse model of high‐fat diet (HFD)‐induced obesity.^20^ Male C57BL/6 obese mice received two intraperitoneal doses (4.2 × 10^7^ cells/kg) of ADMSCs. This stem cell therapy was sufficient to reduce body fat mass in diet‐induced obesity (DIO) animals, despite no change in body weight. In line with these findings also Wang et al. shown that ADMSCs transplantation did not affect HFD‐induced weight gain.^71^ Furthermore, no significant changes in body weight were noted in a diabetic and obese mouse model following Sod2 or catalase (CAT)‐upregulated ADMSCs therapy.^72^ Likewise, in the mouse, NASH model injection of ADMSCs or their small extracellular vesicles (sEVs) did not significantly change body weight and liver‐to‐body weight ratio.^73^ However, these results contradict with other study demonstrating that obese mice treated with brown ADMSCs significantly reduced body weight.^74^ Similarly, ADMSCs infusion significantly suppress the increase in body weight in db/db obese mice and DIO mice.^75^ On the other hand, Cao et al. confirmed the ADMSCs effectiveness, however, isolated from mice not humans, in reducing body weight in DIO animals.^76^ In another study, a single ADMSCs transplantation did not change overall body weight, while a second ADMSCs injection significantly decreased the weight of the obese mice.^77^ Interesting results were provided by the work of Shree et al.^78^ C57BL/6 HFD‐fed mice were administered with human ADMSCs or metformin‐preconditioned ADMSCs. It turned out that mice treated with ADMSCs alone did not change body weight, but significant weight reduction was observed in the metformin‐preconditioned ADMSCs group.^78^ Discrepancies between studies may result from different animal models used in the experiments, distinct adipose tissue depots chosen for ADMSCs isolation, or different protocols used for cells harvesting and culture.

**FIGURE 2:**
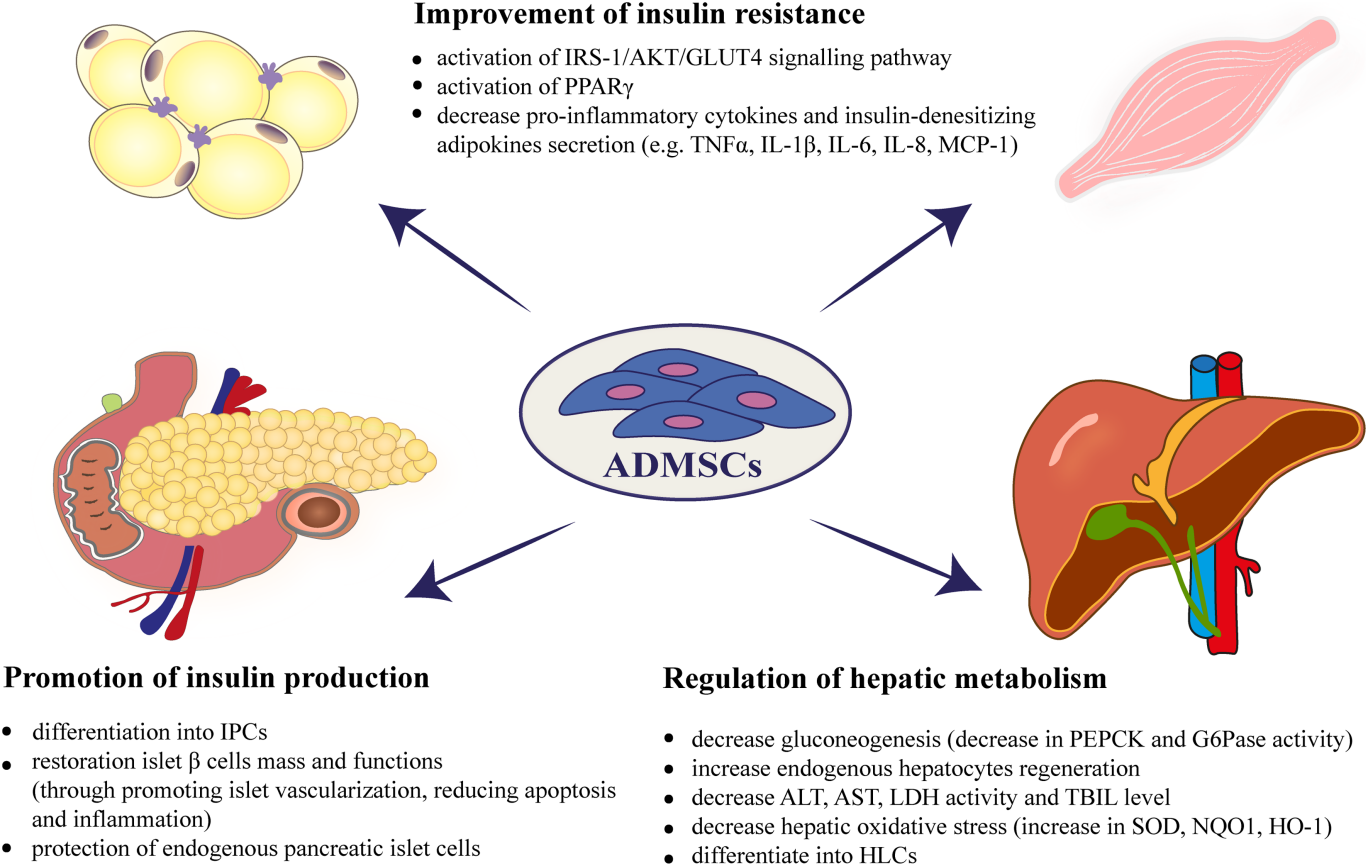
Mechanisms of ADMSCs actions on glucose homeostasis and liver functions. ADMSCs therapy is effective in restoring glycemic status i.e. promotes insulin production and improves insulin sensitivity. Additionally, transplantation of ADMSCs reverses liver steatosis, through reduced inflammation, reduced apoptosis, and improved hepatocyte regeneration. Abbreviations: ADMSCs, adipose‐derived mesenchymal stem cells; AKT, serine/threonine kinase 1; ALT, alanine aminotransferase; AST, aspartate aminotransferase; GLUT4, glucose transporter 4; G6Pase, glucose‐6‐phosphatase; HLCs, hepatocyte‐like cells; HO‐1, heme oxygenase‐1; IL‐1β, interleukin 1β; IL‐6, interleukin 6; IL‐8, interleukin 8; IPCs, insulin producing cells; IRS‐1, insulin receptor substrate 1; LDH, lactate dehydrogenase; MCP‐1, aka CCL2, monocyte chemoattractant protein 1; NQO1, NAD(P)H quinone oxidoreductase 1; PEPCK, phosphoenolpyruvate carboxykinase; PPAR‐γ, peroxisome proliferator‐activated receptor gamma; SOD, superoxide dismutase; TBIL, total bilirubin level; TNF‐α, tumor necrosis factor α

**FIGURE 3:**
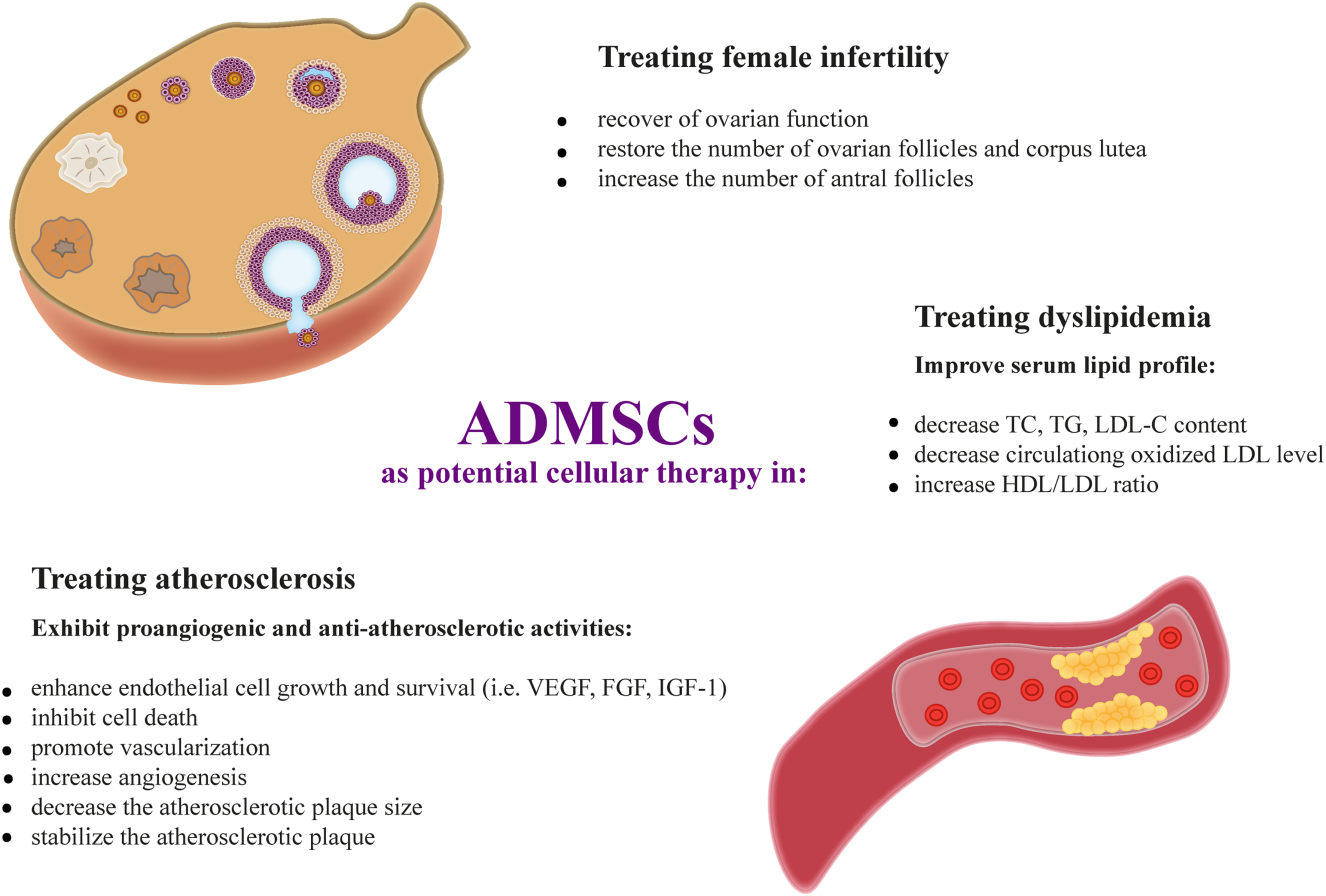
Mechanisms underlying the effects of ADMSCs in improving serum lipid profile, reducing atherosclerosis, and restoring ovarian function. Abbreviations: ADMSCs, adipose‐derived mesenchymal stem cells; FGF, fibroblast growth factor; HDL, high‐density lipoprotein; IGF‐1, insulin‐like growth factor 1; LDL, low‐density lipoprotein; TC, total cholesterol; TG, triglycerides; VEGF, vascular endothelial growth factor

Studies have confirmed that ADMSCs therapy could effectively ameliorate a wide range of obesity‐related comorbidities such as IR, T2DM, women infertility, vascular disorders, NAFLD, and systemic inflammation (Figures 2 and 3, Table 1). The most significant studies are highlighted in this review.

**TABLE 1:** The most relevant preclinical studies pertaining to the therapeutic exploitation of adipose‐derived mesenchymal stem cells (ADMSCs) in the treatment of obesity and its metabolic complications in various animal models

ADMSCs source	Administration route and dose	Animal model	Age	Diet composition	Mean follow‐up period	Body weight and composition	Lipid profile	Glucose, HbA1c levels, GTT	Insulin secretion and insulin sensitivity	Pancreatic islets growth	Pro‐inflammatory and anti‐inflammatory cytokines	Liver function	Author
Human ADMSCs	Intraperitoneal injection of ADMSCs at a dose of 4.2 × 107 cells/kg. A second dose followed after 10 weeks.	Male C57BL/6 mice	9 weeks	Normal chow (9% fat) HFD (60% fat) for 15 weeks	6 weeks after the second injection	Decreased body weight. The percentages of fat mass decreased significantly.	Atherogenic index of plasma (AIP) was significantly reduced.	Significant decrease in plasma glucose level. Positive effect on glycemic status.	Not defined	Not defined	IL‐6 and TNF‐α secretion was decreased.	Not defined	Jaber et al.^20^
Human ADMSCs	Intramuscular injection of ADMSCs suspension at a dose of 5 × 10^5^ cells.	Male C57BL/6 mice	6 weeks	Chow diet (10% kcal from fat) or high‐fat diet (HFD) (60% kcal from fat) for 10 weeks	8 weeks	Body weight did not significantly change.	Decrease in serum triglyceride levels. Reduction in oxidized LDL level.	Decrease in glucose concentration. Improvement in the glucose tolerance.	Reduction in the serum insulin levels. A remarkable decrease in HOMA IR. Increase in the insulin sensitivity.	Not defined	A dramatic reduction in the secreted IL6 cytokine.	Decrease in liver triglycerides.	Shree et al.^21^
Human ADMSCs	5 × 10^5^ ADMSCs injected intramuscularly in the thigh. Additionally, metformin preconditioned ADMSCs (Met‐ADMSCs) were studied.	Male C57BL/6 mice	6 weeks	Chow diet or HFD for 10 weeks	4 weeks	Mice treated with ADMSCs did not show reduction in body weight, whereas Met‐ADMSCs decreased body weight.	TG, oxidized LDL were decreased only in Met‐ADMSCs group.	Met‐ADMSCs decreased fasting glucose level.	Both ADMSCs and Met‐ADMSCs reduced serum insulin levels and increased HOMA IR.	Not defined	Only Met‐ADMSCs treated group showed significant decrease in IL6 levels.	Decrease in IL6 and PAI1 levels in the liver tissue.	Shree et al.^78^
Murine ADMSCs	A single intravenous infusion of ADMSCs at a dose of 2 × 10^6^ cells/ml.	Male C57BL/6 mice	6 weeks	Standard chow diet or high‐fat diet for 20 weeks	2 and 6 weeks after cell infusion	Slightly reduced body weight, but the difference was insignificant.	Decreased TG and increased HDL levels.	Decreased blood glucose level and faster glucose disposal.	Increased	Preservation of β‐cells mass	mRNA expression of F4/80 and TNF‐α was reduced.	Less fat accumulation, suppression of inflammation was observed.	Cao et al.^76^
Human ADMSCs and UC‐MSCs	Intraperitoneally injected ADMSCs (2 × 10^6^ cells), and UC‐MSCs (2 × 10^6^ cells) once a week for 3 weeks.	Male db/db mice with deleted leptin receptor + C57BL/6J mice	6 weeks	Standard diet or HFD for 6 weeks	6 weeks from first ADMSCdelivery	ADMSCs decreased total body weight and adipose tissue weight in db/db and obese mice.	Reduction in TG, TC, LDL‐C levels.	Decrease in fasting blood glucose levels.	Decrease in plasma C‐peptide and glucagon levels. ADMSCs enhanced insulin sensitivity.	Recovery of pancreatic islets. Increase in pancreatic β‐cell mass.	Not defined	Recovery of liver structures. Decrease in IFN‐γ.	Liu et al.^75^
Human brown adipose‐derived MSCs	1 × 10^6^ cells/kg body weight via intraperitoneal injection (every 2 weeks for 10 weeks)	Male C57BL/6J mice	4 weeks	Normal chow diet or HFD for 30 weeks	10 weeks	Decrease in body weight	Decrease in TG, TC. Increase in HDL/LDL ratio	Decrease in fasting glucose level and improved glucose intolerance. Upregulation of GLUT4 in muscle.	Not defined	Not defined	Downregulation of TNF‐α and IL‐4. Upregulation of the anti‐inflammatory cytokines.	Reduction in lipid accumulation in the liver. Decreased serum concentration of AST and ALT, but increased albumin level. Suppression of liver fibrosis and inflammation.	Lee et al.^74^
Human ADMSCs	Systemic transplantation of ADMSCs at a dose of 4.2 × 10^7^ cells/kg body weight	Male B6 mice	7 weeks	Chow diet or HFD for 8 weeks	22 weeks after induction of diabetes	Reduction in body weight.	Not defined	Glucose tolerance and homeostasis were improved	Increased serum level of human insulin. Increase in insulin sensitivity evidenced by GLUT4 upregulation.	Promoted pancreatic islet growth. Downregulation of IL‐1a, IL‐1b and TNF‐1α.	Decrease in circulating TNF‐α and IL‐1 levels.	Reduction in Cpt 1A expression in the liver	Tung‐Qian et al.^77^
Human ADMSCs	Commercially obtained human ADMSCs Intraperitoneal injection of commercially obtained human ADMSCs at a dose of 1.5 × 10^6^; Sod2‐ADMSCs; Cat‐upregulated ADMSCs	C57BL/6J male mice	4–6 weeks	Mice were fed with two different diets: 1) HFD (45% fat) for 14–16 weeks; 2) HFD (60% fat) for 8–10 weeks.	4 weeks post injection	Body weight remained stable. A significant decrease in liver fat content in animals received Sod2‐ or Cat‐ADMSCs.	Not defined	Improved glucose tolerance in studied groups.	Not defined	Not defined	Sod2‐ or Cat‐ upregulated ADMSCs reduce plasma level of TNF‐α.	Reduction in liver triglyceride content.	Domingues et al.^72^
Murine sheet ADMSCs	ADMSCs sheet at a dose 1 × 10^6^ cells/dish were transplanted on the subcutaneous sites of back	MaleC57BL/6J mice	4 weeks	Mice were fed HF/HSD (55% fat, 28% carbohydrate) or normal diet (5% fat, 50% carbohydrate) for 14–16 weeks.	10 days after the surgical procedures.	Decreased	Not defined	ADMSCs sheet transplantation significantly improved glucose intolerance	ADMSCs sheet transplantation improved insulin resistance.	Not defined	ADMSCs sheet transplantation increased and decreased plasma levels of adiponectin and TNF‐α in mice.	Not defined	Ishida et al.^79^
Rat ADMSCs	3 × 10^6^ ADMSCs were injected through the tail vein once a week for 24 weeks.	Male Sprague–Dawley (SD) rats A long‐term T2DM complication rat model.	8 weeks	Normal chow diet (NCD) or a high‐fat diet (HFD; 60% fat) for 8 weeks	24 weeks	Decreased	The level of circulating TGs, TC and LDL‐C was markedly reduced.	Persistent and gradual decrease in blood glucose level. Glucose clearance was improved in the ADMSCs‐treated group.	A marked enhancement in insulin sensitivity after the ADMSCs multiple infusions.	Pancreatic islet function was markedly restored. The ratio of insulin‐positive cells per islet was increased.	Reduction in TNF‐α, IL1β, TGF‐β and increase in anti‐inflammatory molecule IL‐10.	ADMSCs significantly decreased col1 transcripts and TIMP‐1, MMP‐2, 8, and 9.	Yu et al.^80^
Murine ADMSCs	ADMSCs from C57BL/6, db/db, or T2D mice were infused intravenously (5 × 10^5^/mouse via the tail vein.	Male C57BL/6 mice and T2DM mice	C57BL/6–4 weeks old and db/db at 8 weeks of age	Standard chow diet or HFD for a total of 24 weeks	5 weeks after cell infusion	No significant difference in body weight	Not defined	Reduction in blood glucose level	There were no significant differences in plasma insulin levels. Insulin sensitivity was increased.	Ameliorated the destruction to pancreatic islets and restored β‐cell mass.	TNF‐α expression was reduced	ADMSCs infusion reduced liver weight, steatosis and expression of IL‐6, TNF‐a, and F4/80.	Wang et al.^81^
Rat ADMSCs	ADMSCs were injected via tail vein at a dose of 3 × 10^6^ of ADMSCs	Male Sprague–Dawley (SD) rats	8 week‐old	Standard chow diet or HFD (40% fat, 41% carbohydrate and 19% protein) for 8 weeks	24 h after ADMSCinfusion	Not defined	Not defined	Decrease in blood glucose levels. Improvement in glucose homeostasis.	Improvement of insulin sensitivity proved by insulin tolerance tests (IPITT) and HOMA‐IR index value.	Not defined	Not defined	ADMSCs alleviated hyperglycemia	Xie et al.^82^
Murine ADMSCs	ADMSCs (1 × 10^5^ cells) were injected into the spleens of NASH mice.	C57BL/6J mice Nonalcoholic steatohepatitis murine model.	12–14 weeks old	Standard chow diet or atherogenic high‐fat (AT‐HF) diet or HFD for up to 12 weeks	4 weeks	Not defined	Not defined	Not defined	Not defined	Not defined	Decrease in IL‐6, TGF‐β, IL‐23, Acta2, Rorc levels	ADMSCs ameliorate the development of fibrosis during the progression of nonalcoholic steatohepatitis Col4a1 and Col1a1 were significantly downregulated.	Yamato et al.^83^
Rat ADMSCs	Intravenous infusion of ADMSCs through vena caudalis at a dose of 2 × 10^6^/rat	Male Sprague–Dawley rats	8 weeks	Standard chow diet or HFD for 4 weeks	8 weeks	No significant difference in body weight	Not defined	The level of glucose and HbA1c was significantly lower.	ADMSCs infusion improved insulin sensitivity in diabetic rats. ADMSCs treatment did not change serum insulin and C‐peptide levels.	ADMSCs repaired islet cells by reducing the Islet cells apoptosis and promoting their revascularization.	After ADMSCs infusion TNF‐α, IL‐1β and IL‐6 concentrations were lower.	ADMSCs increased GLUT4, phosphorylated IRS‐1, PDK1, and PKCζ expressions in the liver.	Hu et al.^84^

Abbreviations: ADMSCs, adipose‐derived mesenchymal stem cells; AIP, atherogenic index of plasma; ALT, alanine aminotransferase; AST, aspartate aminotransferase; CPT 1A, carnitine palmitoyltransferase 1A; DIO, diet‐induced obesity; GLUT4, glucose transporter 4; GTT, glucose tolerance test; Hb1Ac, glycosylated hemoglobin; HDL, high‐density lipoprotein cholesterol; HF/HSD, high‐fat and high‐sucrose diet; HFD, high‐fat diet; HOMA‐IR, homeostatic model assessment for insulin resistance; IFN‐γ, interferon γ; IL‐10, interleukin 10; IL‐17, interleukin 17; IL‐4, interleukin 4; IL‐6, interleukin 6; IRS‐1, insulin receptor substrate 1; LDL‐C, low‐density lipoprotein cholesterol; MMP‐2, matrix metalloproteinase‐2; MMP‐8, matrix metalloproteinase‐8; MMP‐9, matrix metalloproteinase‐9; NASH, nonalcoholic steatohepatitis; NCD, normal chow diet; PAI1, plasminogen activator inhibitor‐1; PDK1, 3‐phosphoinositide dependent protein kinase‐1; PKC ζ, protein kinase C ζ; TC, total cholesterol; TGF‐β, tumor growth factor β; TGs, triglycerides; TIMP‐1, tissue inhibitor of metalloproteinases 1; TNF‐α, tumor necrosis factor α.

### 4.1 Type 2 diabetes mellitus

#### 4.1.1 Promotion of insulin production

Obesity is associated with the incidence of multiple comorbidities such as diabetes mellitus, a metabolic disease generally classified into type 1 diabetes mellitus (T1DM) or T2DM. Decreased insulin sensitivity in peripheral tissues such as adipose tissue, liver, and skeletal muscles coupled with a progressive loss of adequate insulin secretion is hallmark of T2DM, which accounts for approximately 90%–95% of all diabetes cases. The conventional treatment including lifestyle modifications or pharmacological intervention is only a symptomatic treatment that cannot improve insulin sensitivity or β‐cell dysfunction.^85^ Therefore, a new therapeutic approach is needed to produce significant anti‐diabetic effects and overcome long‐term diabetic complications. Recently, ADMSCs therapy has attracted great attention as a more promising treatment for diabetes due to, inter alia, its capacity to differentiate into insulin producing cells (IPCs). The potential of ADMSCs to derive IPCs was discovered in 2003,^86^ but initially these cells did not secrete insulin. A few years later, Kang et al. developed a method for the differentiation of IPCs to functional endocrine hormone‐producing cells.^87^ Chandra et al. explored the potential of ADMSCs to generate pancreatic hormone‐expressing islet‐like cell aggregates (ICAs) from murine epididymal MSCs.^88^ What is interesting, mature, differentiated ICAs from ADMSCs exhibited enhanced transcript levels of pancreatic endoderm markers like pancreatic and duodenal homeobox gene 1 (Pdx‐1), neurogenin‐3 (Ngn3), paired box 4 (Pax4), NK2 homeobox 2 (Nkx2.2), NK6 homeobox 1 (Nkx6.1), islet 1 transcriptional factor (isl‐1), and insulin.^86^, ^89^, ^90^ Histological analysis showed that ICAs acquire β‐cell features like secretory cells with vacuoles and granules.^88^ The pancreatic‐hormone expressing IPCs transplantation into streptozotocin (STZ)‐induced diabetic mice restored the normoglycemia within two weeks.^88^ Also Lee et at. confirmed that ADMSCs could differentiate into insulin‐producing cells by exogenously expressed the Pdx‐1.^91^ Although Pdx‐1‐induced human ADMSCs reduced blood glucose levels, it did not restore normoglycemia in vivo.^91^ An ex vivo experiment performed on human ADMSCs and their ability to differentiate into insulin secreting cells was completed by Dave et al.^92^ ADMSCs were collected from subcutaneous abdominal AT, cultured in a differentiation medium and analyzed 10 days later.^92^ Interestingly, after this time, ADMSCs began to secrete genes necessary for pancreatic development, that is, Pdx‐1, Pax‐6, and isl‐1.^92^ All three of these factors are essential for the reprogramming nonpancreatic cells to fully functional β cells, in which glucose stimulation leads to the secretion of insulin and C‐peptide. Moreover, Karaoz et al. compared the ability of MSCs derived from adipose tissue and those isolated from bone marrow to differentiate into pancreatic cells.^67^ It turned out that the differentiation potential of ADMSCs into insulin‐producing cells was higher compared with BM‐MSCs. Therefore, ADMSCs could be considered as a preferred cell of choice than BM‐MSCs as their ability to restore metabolic complications of diabetes is better. However, to make stem cell‐based therapy an ideal candidate for clinical implementation, resolution of certain impending issues is needed. For example, neither the long‐term stability of ADMSC‐derived IPCs nor the extent to which in vitro derived IPCs resemble endogenous islets has been identified. What is the minimum number of IPCs to achieve glucose homeostasis to an extent maintained by the endogenous pancreas? Besides, the ADMSCs to IPC differentiation protocol also need to be standardized. In addition to IPC differentiation, ADMSCs promote insulin production by restoring islet function and increasing pancreatic β‐cell mass. These beneficial effects of ADMSCs in pancreatic islet cell repair and regeneration are due to their ability to reduce apoptosis and release paracrine angiogenic factors that promote islet vascularization^84^ (Figure 2).

#### 4.1.2 Improvement of IR

Insulin signaling pathway plays an important regulatory role in adipose and systemic metabolism. The main physiological action of insulin is to stimulate glucose uptake, the rate‐limiting step of postprandial glucose disposal and thus maintaining systemic glucose homeostasis. In adipocytes, insulin‐stimulated glucose uptake is facilitated by the translocation of glucose transporter 4 (GLUT4) to the plasma membrane via inactivating the phosphorylation of TBC1D4 (AS160) and TBC1D1.^93^ Any impairment of insulin effects on target tissues defines IR. In clinical practice, it identifies the reduced effect of insulin on glucose metabolism. Large‐scale population studies have shown that obesity is the most important independent risk factor for the onset and development of IR and further its progression into T2DM.^35^, ^36^ Although many individuals with obesity do not progress to diabetes mellitus, it is generally accepted that two defects are required for progression from IR to T2DM. First, peripheral IR is a primary condition in obesity and a precondition for the onset of type 2 diabetes. Second, dysfunction of β cells to secrete adequate level of insulin to maintain glucose homeostasis is responsible for the progression of IR to a diabetic state.^94^, ^95^ The pathophysiology of IR is complex; although some disturbances in the insulin signaling pathway are known, the mechanism of obesity‐induced IR is not fully understood. Alterations in adipose tissue plasticity are the major trigger of the obesity‐associated metabolic complications. Even though, the number of adipocytes is similar in obese and lean subjects, the adipocytes of patients with obesity and diabetes show necrotic‐like abnormalities.^68^ Overnutrition leads to an enlargement of adipose tissue and an imbalance in the release of pro‐ and anti‐inflammatory cytokines. Hypertrophied adipocytes in subjects with obesity secrete abnormal amount of pro‐inflammatory and insulin‐desensitizing adipokines, including tumor necrosis factor α (TNF‐α), IL‐6, IL‐8, and MCP‐1.^96^, ^97^ Increased secretion of these factors activates many inflammatory signaling pathways, impairs triglyceride (TG) storage, and increases lipolysis, leading to adipose tissue dysfunction. For instance, the activated nuclear factor‐kappa B (NF‐κB) and c‐Jun N‐terminal kinase (JNK) signaling pathways can interfere with insulin tyrosine phosphorylation leading to the development of IR and ultimately T2DM. Besides, reducing local and systemic insulin sensitivity, chronic low‐grade inflammation impairs adipogenesis. In obese animal models, de novo adipogenesis begins when adipocytes reach the critical cell size. These cells are lipid overloaded and insulin resistant; therefore, adipose tissue hyperplasia is a beneficial, adaptive response to overnutrition that can prevent metabolic alterations in a chronic state of positive energy balance.^34^ Moreover, hypertrophied adipocytes are characterized by an increased leakage of FFAs, taken up by other peripheral tissues, which causes ectopic lipid accumulation and lipotoxicity.^98^ Therefore, adipocytes hypertrophy may cause and exacerbate IR by both inflammation‐dependent and inflammation‐independent mechanisms.

The effectiveness of ADMSCs cell‐based therapy in the maintaining glucose homeostasis, alleviation IR, and treatment of T2DM has been investigated by several studies.^81^, ^84^, ^85^, ^99^ So far, it has been shown that multiple administration of ADMSCs upregulated GLUT4 expression in adipose and skeletal muscle tissues and reduced gluconeogenesis in the liver of DIO mice, improving glucose intolerance.^74^ In addition, cell therapy exerts anti‐obesity and anti‐diabetic effects by activating peroxisome proliferator‐activated receptor gamma (PPARγ), a master transcriptional regulator of adipogenesis, and its downstream target genes controlling fatty acid synthesis, esterification, and sequestration TG within lipid droplets of adipocytes^74^ (Figure 2). Other researchers demonstrated that ADMSC sheets transplantation into the subcutaneous sites improved glucose intolerance in mice fed with high‐fat and high‐sucrose diet (HF/HSD).^79^ Furthermore, Shree et al. examined the therapeutic effect of secretome from conditioned media (CM) of ADMSCs for the treatment of T2DM.^99^ They showed that CM of ADMSCs stimulates glucose uptake by enhancing GLUT4 expression and phosphorylation of Akt protein and reduces IL‐6 and plasminogen activator inhibitor 1 (PAI1) levels in both 3T3L1 and C2C12 insulin‐resistant cells.^99^ Moreover, 3T3L1 preadipocytes treated with ADMSC‐CM did not differentiate into mature adipocytes, which suggest that these cells can be used in prevention of obesity and further development of IR.^99^ As a general observation, ADMSCs alleviate IR via reducing oxidative and inflammatory cellular stress. Indeed, ADMSCs and their conditioned medium inhibited the inflammatory response, as evidenced by a reduction in intracellular production of reactive oxygen species (ROS), endoplasmic reticulum stress markers (CHOP1, IRE1), and the expression of oxidative and inflammatory stress‐induced serine kinases (SISK), including inhibitory‐κB kinase (IKKβ), JNK, extracellular signal regulated kinases (ERK), and ribosome protein subunit 6 kinase 1 (S6K1) in insulin‐resistant 3T3‐L1 adipocytes and C2C12 myotubes.^85^ In another study, Wang et al. compared the therapeutic properties of ADMSCs isolated from HFD/STZ‐induced T2DM and the leptin receptor‐deficient (db/db) mice with cells from healthy C57BL/6 mice.^81^ Although the multipotency of ADMSCs in studied groups was comparable with control animals, significant decrease in proliferation rate was observed. Nevertheless, single intravenous infusion of ADMSCs derived from T2DM or db/db mice increased insulin sensitivity, improved glucose tolerance, and reduced inflammation in T2DM mice 5‐week posttransplantation.^81^ These ADMSCs acquired from T2DM mice still show significant insulin‐sensitizing and pancreatic protective effects and can therefore be used in the treatment of IR and T2DM. Furthermore, transplantation of insulin‐secreting cells differentiated from human eyelid ADMSCs into T2DM mice model significantly increased circulating insulin and C‐peptide levels and ameliorated hyperglycemia in the studied animals. Additionally, T2DM mice group exhibited lower serum levels of TGs, FFAs, and IL‐6.^100^ Regulation of hepatic glucose metabolism by ADMSCs was examined by Xie et al. on a T2DM rat model. Diabetic rodents were able to lower blood glucose level, increase glucose tolerance, and improve insulin sensitivity 3 h after ADMSCs injection, and the effect continued up to 24 h after cells infusion.^82^ Moreover, ADMSCs administration reduced the expression of the gluconeogenesis rate‐limiting enzymes, that is, phosphoenolpyruvate carboxykinase (PEPCK), and glucose‐6‐phosphatase (G6Pase) in hepatocytes of diabetic animals.^82^

Although ADMSCs therapeutic effect is astounding in the treatment of early phase T2DM, there are few reports on the late phase of diabetes. For instance, Hu et al. induced T2DM in a rat model that mimics long‐term complications of diabetes.^84^ ADMSCs infusion was done at the 28th day after STZ injection and effectively ameliorated hyperglycemia in T2DM rats. In parallel, ADMSCs treatment restored pancreatic islet β‐cell function and improved IR via upregulation of GLUT4 and enhancing IRS‐1 phosphorylation in insulin‐sensitive tissues.^84^ The same phenomenon was observed by Yu et al.; in this study, multiple intravenous infusions of ADMSCs (weekly for 24 weeks), were effective in restoring glucose homeostasis, ameliorating IR, and altering the progression of metabolic complications in a rat model of long‐term T2DM complications.^80^ It seems that multiple ADMSCs infusions, rather than a single infusion or several infusions have therapeutic potential in the treatment of advanced stage of T2DM. In overall, available studies proved that treatment with ADMSCs has an anti‐diabetic effect and alleviates not only the early stages but also the long‐term complications of diabetes such as chronic kidney disease, pulmonary fibrosis, liver fibrosis or steatosis, cardiovascular complications, and female infertility.

### 4.2 Female infertility

Female infertility is a global medical condition, and most infertile women exhibit metabolic aberrations like IR, T2DM, or dyslipidemia, which are often aggravated by concomitant abdominal obesity or frank obesity. Recent new data have shown that ADMSC‐based therapy has also potential effect for recovery of ovarian function in women with obesity. Obese women in particular suffer from menstrual disorders leading to anovulation and infertility. Scientists have demonstrated that ADMSCs contribute to angiogenesis and restore the number of ovarian follicles and corpus lutea in ovaries^101^ (Figure 3). Terraciano et al. found improvement in ovarian function after chemotherapy using a preclinical mouse model.^102^ Despite high therapeutic potential of ADMSCs in the treatment of the most common disorders leading to female infertility, ADMSCs therapy should not be considered for endometriosis. Indeed, allogenic ADMSCs from women with endometriosis increased the growth of ectopic endometrial tissue and thus promote the development of endometriosis in vitro.^103^ ADMSCs were used also to restore ovarian function in a rat model of premature ovarian insufficiency (POI). Stem cells injection increased the number of antral follicles and the pregnancy rate increased from 50% (control group) to 72.7% (ADMSCs group).^104^ Although ADMSCs therapy might be a potential treatment strategy for female infertility, the current research is in preclinical phase or at a very early clinical trial phase.

### 4.3 Dyslipidemia and atherosclerosis

Dyslipidemia is characterized by elevated levels of serum TGs, total cholesterol (TC), low‐density lipoprotein cholesterol (LDL‐C), and low levels of HDL‐C.^105^ There is strong positive correlation between increased BMI, WAT capacity, and dyslipidemia. Importantly, hyperlipidemia is one of the major risk factors for CVD, the leading cause of morbidity and mortality worldwide, through the development and progression of atherosclerosis.^106^ One of the first stages of the atherosclerotic process is the aggregation of atherosclerotic lipoprotein particles in the compliant wall of the coronary artery, mainly small dense LDL and oxidized LDL, followed by a reactive inflammatory process, smooth muscle cell (SMC) proliferation, fibrosis, and calcification.^107^ Therefore, the controlling serum lipids profile is a cornerstone in the prevention and treatment of atherosclerotic CVD. Despite lifestyle changes, patients with dyslipidemia and atherosclerosis need to follow complex medication regimens. Statins and fibrates are the most commonly used treatments with the high pharmacological efficiency to reduce TC, TG, LDL, and serum concentrations.^108^ However, several adverse effects and limitations are associated with their use. For example, they cannot be taken by women of childbearing age (without contraception), breastfeeding women, with impaired liver function and patients under 10 years of age.^109^ Recently, cellular therapy using ADMSCs has offered the potential to normalize hyperlipidemia and associated CVDs, which is related to their ability to improve serum lipid profile, promote angiogenesis, and inhibit cell apoptosis (Figure 3). This beneficial anti‐atherosclerotic effects of ADMSCs are due to their robust secretion of paracrine factors that enhance endothelial cell growth and survival. Indeed, these stem cells release a large number of growth factors like vascular endothelial growth factor (VEGF), fibroblast growth factor (FGF), hepatocyte growth factor (HGF), IGF‐1, stromal cell‐derived factor 1, as well as proangiogenic cytokines, which makes them an ideal candidate for angiogenic therapy.^110^, ^111^, ^112^ Recently, Liu et al. compared the reparative/regenerative capacity of ADMSCs with umbilical cord‐derived MSCs (UC‐MSCs). The human ADMSCs were isolated from abdominal SAT and administered once a week for 3 weeks into DIO mice. The authors have reported that ADMSCs, but not UC‐MSCs, could significantly improve serum lipid profiles by reducing TC, TGs, and LDL‐C levels in DIO mouse model. In addition, histological analysis of adipose tissue revealed that the size of abdominal adipocytes was markedly decreased, which was not observed in mice treated with UC‐MSC.^75^ Apart from that, Lee et al. have compared the effectiveness of several methodological approaches in the treatment of obesity and its associated MSs.^74^ They treated obese mice with human MSC, MSC‐derived brown adipocytes, and MSC lysate repeatedly for 10 weeks and showed that all three MSC‐based therapies improved obesity‐related MSs. Nevertheless, transplantation of only ADMSCs showed the strongest beneficial effect; it reduced body weight, decreased TGs and cholesterol, and increased HDL/LDL ratio in obese mice.^74^ Similarly, Yu et al. proved the effectiveness of ADMSCs in improving the serum lipid profile in a rat model of long‐term complications of T2DM.^80^ On the other hand, preconditioning of ADMSCs with metformin have a better therapeutic value in ameliorating IR and reversing type 2 diabetes than a single infusion of stem cells. Out of the three groups (metformin, ADMSCs, and metformin ADMSCs), metformin‐ADMSCs treated group showed a remarkable decrease in serum TG level and circulating oxidized LDL, a marker of oxidative stress, in DIO mice.^78^ These pieces of evidence show that preconditioning of ADMSCs exerts synergistic action in counteracting dyslipidemia and thus may improve therapeutic potential of ADMSCs.

It is well known that hyperlipidemia is not only a risk factor for endothelial dysfunction and inflammatory response in the development and progression of vascular disease, but also induces alterations in MSCs. MSCs have the ability to migrate from media or adventitia to the intima, where they may differentiate into different cell types and secrete beneficial factors that reduce inflammation and restore endothelial function.^113^ It has been shown that MSCs can stabilize the plaque by differentiating into SMCs and thus reduce the complication of atherosclerosis, including plaque rupture (Figure 3). Recently, ADMSCs has been explored as an attractive therapeutic approach for the treatment of atherosclerosis. Because intravascular MSC administration presents a risk of vascular occlusion, conditioned medium from ADMSCs attracts more attention. Takafuji et al. found that humoral factors secreted from ADMSCs ameliorate atherosclerosis in low‐density lipoprotein receptor‐deficient (LDLR^−/−^) mice.^114^ The conditioned medium from ADMSCs was intravenously injected twice a week for 13 weeks in HFD fed animals. Despite ADMSC‐CM did not affect serum lipid profiling in HFD‐fed animals, it decreased the atherosclerotic plaque area in the aorta (by 41%) and aortic root (by 30%) by suppressing the accumulation of cell adhesion molecules (CAMs) and macrophages in the vascular walls.^114^ Moreover, ADMSCs also have the ability to influence the phenotypes of macrophages that regulate the structure and function of blood vessels. As macrophages have multifunctional roles, they show different phenotypes, M1 and M2 pro‐inflammatory and anti‐inflammatory, respectively. ADMSC‐CM cytokines promoted M2 polarization by activating the signal transducer and activator transcription 3 (STAT3) pathways and inhibiting the MAPK and NF‐κB pathways.^114^ Using the cell culture model, murine bone marrow‐derived macrophages were incubated with ADMSCs or with conditioned medium from ADMSCs, and interestingly both types of intervention were able to reprogram macrophages to regulatory/M2‐like phenotype.^115^ To further established regulatory effects of ADMSCs on macrophages, Souza‐Moreira et al. have determined the molecular mechanism of lipid droplet formation in macrophages using conditioned medium from ADMSCs.^116^ They revealed that paracrine factors released by ADMSCs promotes lipid droplet biogenesis via mTOR Complex 1 (mTORC1) and 2 (mTORC2) and macrophage polarization as indicated by increased IL‐10 secretion and nitric oxide (NO) release. These findings suggest that ADMSC may hold therapeutic potential for dyslipidemia and associated CVDs, but the major obstacle is that successful results come only from animal models or in vitro studies.

### 4.4 Immunomodulatory and anti‐inflammatory effects of ADMSCs in reliving inflammation

Scientists in the early 1990s discovered the relationship between elevated levels of several pro‐inflammatory cytokines and excess adiposity.^117^ Obesity induces a state of low‐grade chronic inflammation, which is a hallmark of the infiltration of immune cells in adipose tissue and the production of pro‐inflammatory cytokines and chemokines. The deregulated inflammatory pathway leads to adipose tissue dysfunction, essential in the pathogenesis of metabolic complications of obesity. Because the acquisition of ADMSCs is relatively simple and much less invasive compared to other MSCs, their clinical use is expected. The growth factors, chemokines, and anti‐inflammatory cytokines secreted from ADMSCs are responsible for their beneficial effects. Importantly, ADMSCs are able to adopt either anti‐inflammatory or pro‐inflammatory phenotype by interacting with the immune cell in its microenvironment. This is crucial for understanding their therapeutic potential in immune‐mediated disorders.^118^, ^119^ In the absence of an inflammatory conditions (insufficient levels of INF‐γ and TNF‐α), human and mice MSCs exhibit pro‐inflammatory phenotype and secrete chemokines (e.g., macrophage inflammatory protein 1α/β [MIP‐1α/β], RANTES, chemokine [C‐X‐C motif] ligand 9 [CXCL9], and ligand 10 [CXCL10]) to activate T cells. MSCs also promote proliferation and activation of M1 macrophage that express INF‐γ and TNF‐α.^120^ On the opposite, in the presence of inflammatory environment, MSCs suppress the immune response through producing high levels of cytokines, including TGF‐β, HGF, and soluble factors like indoleamine 2,3‐dioxygenase (IDO), prostaglandin E2 (PGE2), IL‐10, heme oxygenase‐1 (HO‐1), TNF‐inducible gene‐6 protein (TSG6), or NO that suppress T‐cell proliferation and directly promote the activation of regulatory T cells (Tregs).^121^ Furthermore, MSCs stimulate macrophages to produce anti‐inflammatory and immunosuppressive cytokines like as IL‐10 and TGF‐1β and thereby promote polarization towards anti‐inflammatory M2 phenotype. Polarized M2 macrophages produce IL‐10, TGF‐β, and chemokine ligand‐18 (CCL18) that favor the emergence of Tregs, suppress immune response, and dampen inflammation^122^ (Figure 1). Heo et al. have found that exosomes derived from ADMSCs acted as mediators and induced M2 polarization in vitro.^123^ Under pathological conditions, these cells are mobilized towards inflammatory signals and damaged tissues, which make them an excellent therapeutic vector. In addition to that, ADMSCs have been shown to inhibit immunoglobulin production and inhibit the differentiation and maturation of monocyte‐derived dendritic cells (mDCs) stronger than BM‐MSCs.^124^ Several reports have demonstrated that ADMSCs can secrete an array of anti‐apoptotic, mitogenic, and growth factors, such as IGF‐1, VEGF, FGF, epidermal growth factor (EGF), platelet‐derived growth factor (PDGF), TGF‐β, and HGF. Many of which are produced in response to TNF‐α, lipopolysaccharide (LPS) or hypoxia by a NF‐κB, p38 mitogen‐activated protein kinase (MAPK), and STAT3‐dependent mechanisms.^125^, ^126^, ^127^ Thus, ADMSCs might modify the organ microenvironment preventing the apoptosis.

Transplanted ADMSCs not only improved the inflammatory environment but also exerted anti‐oxidant effects. In subjects with obesity, increased production of ROS leads to systemic oxidative stress, mainly due to reduced antioxidant capacity, contributing to the development of obesity‐related diseases. In a mouse model of obesity, a single intravenous infusion of ex vivo expanded syngeneic ADMSCs suppress inflammation in insulin‐targeting tissues such as liver and adipose tissue. At the molecular level, obese mice treated with ADMSCs had reduced expression of IL‐6 and F4/80 in the liver, and such protective effects of stem cell persisted at least 6 weeks after transplantation.^76^ In the same model, Jaber et al. showed a decrease in TNF‐α and IL‐6 serum levels in obese mice treated with ADMSCs to concentrations similar to that of lean mice.^20^ It has been demonstrated that not only human ADMSCs but also their conditioned medium or their cell lysate are able to lower PAI1 and ApoB levels in the liver and circulating IL‐6 and oxidized LDL in DIO mice.^21^ Furthermore, Liao et al. revealed that the intravenous administration of ADMSCs significantly decreased serum levels of TNF‐α, IL‐6, and C‐reactive protein (CRP) in a type 2 diabetic rat model with liver fibrosis.^128^ Subsequently, in a rat model that closely mimic the long‐term metabolic complication found in T2DM, multiple (once a week for 24 weeks) ADMSCs administrations attenuated systemic inflammation. The downregulation of pro‐inflammatory cytokines, such as IL‐6, IL‐1β, and TNF‐α, and upregulation of anti‐inflammatory cytokine, IL‐10, after ADMSCs infusions has also been reported. Other researchers have shown that in vitro preconditioning of ADMSCs to prepare for a harsh environment in vivo increases their immunotherapeutic capacity as evident via immunosuppressive and immunomodulatory functions. Following this concept, ADMSCs were cultured for 48 h in either normoxia (21% O_2_) or hypoxia (2% O_2_) with or without the addition of Cytomix that contains TNF‐α, INF‐γ, and IL‐1β to mimic an injurious inflammatory milieu.^129^ These preconditioning approaches increased the expression of key anti‐inflammatory genes such as IL‐8, IL‐1 receptor antagonist (IL‐1RA), and enhanced suppression of T‐cell proliferation, facilitating immunomodulatory and anti‐inflammatory properties. Although this preconditioning method may be valuable for producing ADMSCs for anti‐inflammatory indications, there are some limitations that should be considered prior to clinical application. First, ADMSCs showed tissue factor (TF) upregulation in the Cytomix‐hypoxic group; hence, thromboembolism could have significant clinical consequences. Second, the combination of Cytomix and hypoxia impairs the differentiation capacity of preconditioned ADMSCs, although Cytomix alone treated ADMSCs cultured under normoxia retain their multipotential and self‐renewing properties and showed augmented functions.^129^ In another recent study, transplantation of the ADMSCs sheets reduced the levels of TNF‐α in both plasma and VAT in mice fed HF/HSDs. The authors demonstrated that MSCs sheet transplantation is a superior method of cell delivery compared with conventional cell transplantation in which deconstruction of the cell‐to‐cell junction and the extracellular matrix leads to poor cell engraftment and cell survival.^79^ The results of recent studies highlight the ADMSCs efficacy in alleviating chronic inflammation, restoring oxidative balance and promoting the repair of damaged tissues in the progression of obesity and related comorbidities (Figure 2). However, most of the available studies are derived from animal models, as some differences between human and rodents MSCs exist it should be taken into consideration before translating their effects into clinical application. A notable example of these differences is the use of diverse soluble mediators, including inducible NO synthase (iNOS) for mice and IDO for humans to suppress T‐cell proliferation.^130^

### 4.5 Nonalcoholic fatty liver disease

NAFLD ranges from simple steatosis to more progressive NASH with active hepatocellular necrosis, liver inflammation, and tissue damage, to cirrhosis, and in some cases hepatocellular carcinoma (HCC).^131^, ^132^ NAFLD is characterized by presence of steatosis in more than 5% of hepatocytes with or without mild lesions.^56^, ^133^ Although NAFLD is highly prevalent in the general population, most of the affected patients mainly suffer from simple, non‐life‐threatening steatosis, while 5%–10% of NAFLD patients actually develop NASH. The liver failure is highly associated with the incidence of severe obesity, and MS.^131^ It affects 75%–90% of patients with obesity or morbid obesity compared to general population.^134^ The underlying pathogenesis of NAFLD is multifactorial and complex. However, among the most important NAFLD inducers are accumulation of hepatic lipids, ER/oxidative stress, pro‐inflammatory cytokines, mitochondrial dysfunction, and genetic and epigenetic factors.^135^ Although the pathogenesis of NAFLD is fairly well understood, there is no satisfactory therapeutic treatment of this disease.

ADMSCs with high multilineage potential, self‐renewal capacity, and anti‐inflammatory and anti‐oxidant properties have attracted great attention as a promising means of liver regeneration. Several lines of evidence indicated that ADMSCs can readily differentiate into hepatocyte‐like cells (HLCs) and adopt the hepatocyte phenotype.^136^, ^137^, ^138^ Notably, these ADMSCs‐derived HLCs possess the functional properties of mature hepatocytes, including albumin secretion, urea formation, glycogen synthesis, LDL uptake, cytochrome P450 (CYP) enzyme activity, and expression of carbamoyl phosphate synthetase.^136^ Transplantation of HLCs attenuated postoperative acute liver failure (ALF) in a rat model of 90% liver resection by normalizing the levels of amino acids, acylcarnitines, sphingolipids, and glycerophospholipids, reducing the rate of apoptosis and increasing the rate of proliferation.^137^ Banas et al. demonstrated that ADMSC‐derived HLC restores liver functions such as ammonia and purine metabolism and reduces markers of liver injury such as alanine aminotransferase (ALT), aspartate aminotransferase (AST) in mice with ALF.^138^ In the another study, transplantation of HLCs preserved liver functions (via the secretion of IL‐10, IL‐6, and TGF‐β) and prolonged the survival of mice with carbon tetrachloride (CCl_4_)‐induced liver injury.^139^ On the other hand, the use of HCLs for liver failure has also been questioned by studies that found that transplanted MSCs, but not HCLs were more effective in regaining liver function.^140^, ^141^ Although HLCs can improve liver function in vitro, these immature hepatocytes simply progress to the cell death pathway, whereas ADMSCs are not so sensitive to the damaged microenvironment. Thus, autologous transplantation of ADMSCs in vivo is recommended for liver regeneration.

There is evidence that secreted chemotactic cytokines and inflammatory factors from injured liver tissue or hepatocytes attract ADMSCs to the lesion site, supporting hepatocyte proliferation as well as endogenous hepatocytes regeneration. According to the current state of knowledge, ADMSC engraft into recipient livers contributes to the liver regeneration and the maintenance of its function without eliciting an immune response in vivo. Importantly, ADMSCs also exhibit antioxidant properties by increasing the enzymatic activity of the antioxidant defense such as superoxide dismutases (SODs), glutathione peroxidases (GPx), CATs, and the levels of other direct antioxidants such as glutathione (GSH).^142^ In one of the experimental approach, ADMSCs transplant in vivo restored the oxidative balance by reducing the content of malondialdehyde (MDA) serum level and increased the level of GSH and the activity of CATs in the liver damaged with methotrexate.^143^ These results are concordant with other studies demonstrating that ADMSC therapy decreased hepatic oxidative stress via upregulation the activity of SOD, NAD(P)H quinone oxidoreductase 1 (NQO1), HO‐1, and downregulation of myeloperoxidase (MPO) following ischemia/reperfusion injury of the liver.^144^, ^145^ Furthermore, it has been demonstrated that ADMSCs suppress hepatic inflammation, which is important in the progression of NAFLD. Accordingly, Yamato et al. showed that ADMSCs transplantation ameliorated fibrosis and hepatic malfunction through reducing the number of IL‐17‐secreting hepatic inflammatory cells (HICs) in the murine NASH model.^83^ Moreover, in severe liver fibrosis, ADMSCs administration downregulated the expression of pro‐inflammatory cytokines (TNF‐α, IL‐4), while increased the expression of anti‐inflammatory cytokines.^74^

Several studies have shown that infusion of ADMSCs can significantly reduce markers of liver injury such as ALT, AST, total bilirubin (TBIL), and lactate dehydrogenase (LDH) while increasing albumin and maintaining prothrombin activity in murine models of cirrhosis or liver fibrosis.^133^, ^146^, ^147^ Some researchers have found that the route of ADMSCs administration affects the effectiveness of stem cells. To test this hypothesis, Kim et al. administered ADMSCs via the tail vein, portal vein and directly to liver parenchyma in C57BL/6 mice with liver injury induced by CCl_4_.^148^ Transplantation of ADMSCs into the systemic circulation most effectively amended the injured liver, as evidenced by the reduction in the amount of pro‐inflammatory cytokines and an increase in anti‐inflammatory cytokines in the animal model. Consequently, other researchers have shown that ADMSC transplantation slows the progression of NAFLD.^149^, ^150^ A study on HFD‐fed mice showed a decrease in markers of liver injury (e.g., LDH, AST, ALT, and TBIL), steatosis, and inflammatory factors after ADMSCs infusion.^72^ Although the animals did not restore liver function to the level of a healthy individual, they still showed inhibition of NAFLD development.^72^ In another study, serum levels of TBIL and AST decreased significantly 2 weeks after an infusion of ADMSCs, although these changes were not detected 4 weeks after transplantation. The authors speculate that the therapeutic effects of ADMSCs were partially attenuated by continuing to feed HFD during the experiment.^45^ Recently, the therapeutic potential of ADMSCs and their sEVs, which possess immunomodulatory activities, were assessed in melanocortin type‐4 receptor knockout (Mc4r‐KO) NASH mouse model.^73^ Interestingly, ADMSCs and sEVs demonstrated anti‐inflammatory and anti‐fibrotic effects to a similar extent. Histological analysis showed that both of treatments had no effect on fat accumulation, but improvement in liver fibrosis was still observed.^73^ In addition, ADMSCs transplantation reduced liver fibrosis through reducing type 1 collagen fibers in the analyzed hepatocytes together with a decrease in tissue inhibitor of metalloproteinases‐1 (TIMP‐1) and matrix metalloproteinase‐2 (MMP‐2) in T2DM rats with NASH‐like features.^80^ Similarly, Liao et al. found that ADMSCs could ameliorate liver fibrosis in rats with type 2 diabetes. They noticed that the TGF‐β1//mothers against decapentaplegic homolog 3 (SMAD3) signaling pathway, which plays an important role in the progression of liver fibrosis, was downregulated after ADMSCs transplantation in the fibrotic liver tissues.^128^ Altogether, the reported data indicate that ADMSCs ameliorate the development of chronic liver disease, including NAFLD, liver fibrosis and cirrhosis in animal models and may account for their broad therapeutic efficacy in the clinical treatment of liver disease.

The safety and efficacy of ADMSCs in the treatment of chronic liver failure have been demonstrated in a phase I clinical trial. Autologous freshly isolated ADMSCs with the maximal number of cells 6.6 × 10^5^ cells/kg were injected into the common hepatic artery of four patients with liver cirrhosis.^151^ No serious adverse effects were observed during the 1‐month study period. All patients had upregulated the expression of liver regeneration‐related factors (HGF and IL‐6) and exhibited improved synthetic liver function (prothrombin time, albumin) at follow‐up.^151^ This clinical study demonstrates the therapeutic efficacy of ADMSCs in maintaining liver function in patients with liver cirrhosis.

## 5 CONCLUSIONS

**FIGURE 4:**
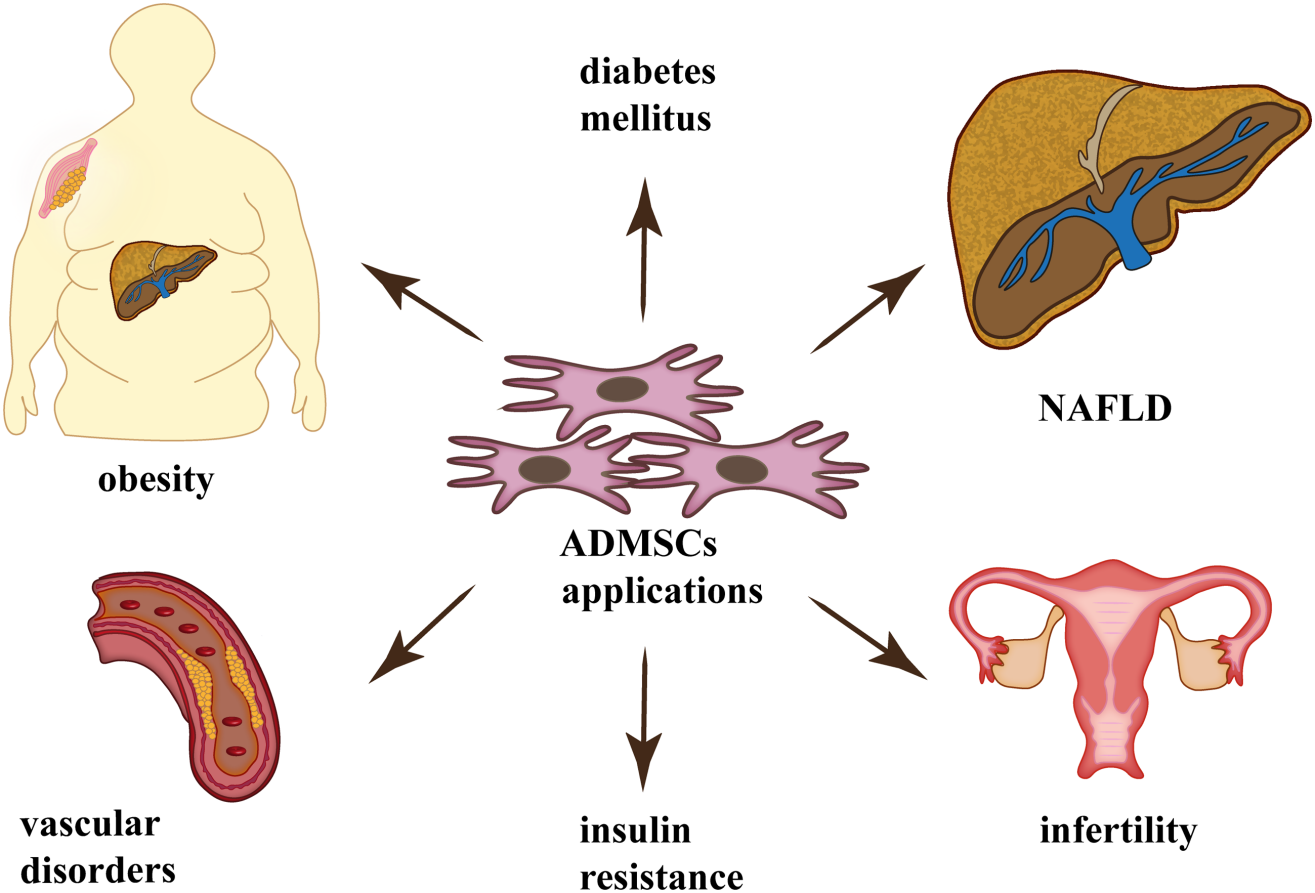
Potential therapeutic application of ADMSCs in the treatment of obesity and related comorbidities such as diabetes, insulin resistance, vascular disorders, infertility, and NAFLD. Abbreviations: ADMSCs, adipose‐derived mesenchymal stem cells; NAFLD, nonalcoholic fatty liver disease

ADMSCs are promising candidates for cell‐based therapy due to their abundancy, ease of isolation, multilineage potential, self‐renewal capacity, anti‐apoptotic, anti‐oxidative, and anti‐inflammatory properties. Growing evidence points to the therapeutic potential of ADMSCs in the treatment of obesity‐related metabolic complications such as T2DM, IR, hepatic steatosis, fibrosis, infertility, vascular disorders, and systemic inflammation (Figure 4). The mechanisms by which ADMSCs exert beneficial effects include their ability to differentiate into specific cells, that is, HLCs, IPCs, as well as the release of a broad spectrum of biomolecules that can restore liver, pancreatic islet β cell, and endothelial function, improve IR, and subsequently promote the suppression of apoptosis, inflammation and ROS production (Figures 2 and 3). Importantly, no animal deaths or any serious adverse events were reported following ADMSCs injection in the tested models. Success in preclinical research has led to the progression to clinical trials. Majority of the clinical trials conducted so far have used autologous ADMSC, which minimize the risk to recipients. Indeed, very few serious adverse events were reported. Nevertheless, it is known that ADMSCs acquired from patients with chronic inflammatory diseases like obesity are less effective in immunomodulation compared with lean, metabolically healthy individuals. Because health characteristics and age of the donor impact on the ADMDCs properties, allogenic ADMSCs may provide more effective cellular therapy. Up to date, only a few randomized, controlled trails have been performed to assess the therapeutic potential of these cells in alleviating metabolic complications of obesity. Some of the major problems that still need to be resolved concern to the standardization of cell processing and culture protocols, ideal transplant route, dosing, and timing of ADMSCs administration. Systemic infusion appears to be more effective, while peripheral intravenous injections are easier to handle and have fewer side effects. Likewise, the dosage varies from study to study; in some cases, a single injection was not sufficient; therefore, multiple injections were administered. Furthermore, it should be taken into account that ADMSCs obtained from different anatomical regions of adipose tissue may vary in their functions and biological properties. Another aspect is the expansion of stem cells into multiple passages, which may begin to undergo replicative senescence leading to genetic instability and thus reducing therapeutic efficacy. Resolving these issues is expected to result in a more rational exploitation of their therapeutic use. Overall, ADMSCs represent a potential strategy for treating the metabolic complications of obesity, but large‐scale and controlled trails are required to confirm efficacy before their widespread clinical application.

## ACKNOWLEDGMENTS

This study was supported by the National Science Center (grant no. 2016/23/D/NZ3/01660) and the Medical University of Bialystok (grant no. SUB/1/DN/20/010/1118).

## CONFLICT OF INTEREST

The authors declare no conflict of interest.

## AUTHOR CONTRIBUTIONS

AM created the concept, collected the data, and designed the outline. AM and BEN prepared a draft of the manuscript that was revised by AC. All authors contributed to the article and approved the submitted version.
